# Balancing selection, genetic drift, and human‐mediated introgression interplay to shape MHC (functional) diversity in Mediterranean brown trout

**DOI:** 10.1002/ece3.7760

**Published:** 2021-07-15

**Authors:** Lorenzo Talarico, Silvio Marta, Anna Rita Rossi, Simone Crescenzo, Gerardo Petrosino, Marco Martinoli, Lorenzo Tancioni

**Affiliations:** ^1^ Laboratory of Experimental Ecology and Aquaculture Department of Biology University of Rome “Tor Vergata” Rome Italy; ^2^ Department of Environmental Science and Policy University of Milan Milan Italy; ^3^ Department of Biology and Biotechnology C. Darwin University of Rome “La Sapienza” Rome Italy; ^4^ Consiglio per la Ricerca in Agricoltura e l'Analisi dell'Economia Agraria (CREA) Centro di Zootecnia e Acquacoltura Monterotondo Italy

**Keywords:** body condition, hybridization, major histocompatibility complex, MHC supertypes, rare allele advantage, *Salmo trutta* complex

## Abstract

The extraordinary polymorphism of major histocompatibility complex (MHC) genes is considered a paradigm of pathogen‐mediated balancing selection, although empirical evidence is still scarce. Furthermore, the relative contribution of balancing selection to shape MHC population structure and diversity, compared to that of neutral forces, as well as its interaction with other evolutionary processes such as hybridization, remains largely unclear. To investigate these issues, we analyzed adaptive (MHC‐DAB gene) and neutral (11 microsatellite loci) variation in 156 brown trout (*Salmo trutta* complex) from six wild populations in central Italy exposed to introgression from domestic hatchery lineages (assessed with the LDH gene). MHC diversity and structuring correlated with those at microsatellites, indicating the substantial role of neutral forces. However, individuals carrying locally rare MHC alleles/supertypes were in better body condition (a proxy of individual fitness/parasite load) regardless of the zygosity status and degree of sequence dissimilarity of MHC, hence supporting balancing selection under rare allele advantage, but not heterozygote advantage or divergent allele advantage. The association between specific MHC supertypes and body condition confirmed in part this finding. Across populations, MHC allelic richness increased with increasing admixture between native and domestic lineages, indicating introgression as a source of MHC variation. Furthermore, introgression across populations appeared more pronounced for MHC than microsatellites, possibly because initially rare MHC variants are expected to introgress more readily under rare allele advantage. Providing evidence for the complex interplay among neutral evolutionary forces, balancing selection, and human‐mediated introgression in shaping the pattern of MHC (functional) variation, our findings contribute to a deeper understanding of the evolution of MHC genes in wild populations exposed to anthropogenic disturbance.

## Graphical abstract

We investigate evolutionary mechanisms shaping genetic structure and diversity at the major histocompatibility complex class II gene (MHC‐DAB) in wild populations of Mediterranean brown trout (*Salmo trutta* L. complex) exposed to introgression from domestic (hatchery) lineages. We reveal the role of historical positive selection and current balancing selection (rare‐allele advantage, but not heterozygote advantage or divergent allele advantage) to mantaining high MHC (functional) polymorhism. Also, we provide evidence for the interplay between genetic drift and human‐mediated introgression to shape MHC diversity and population structure.

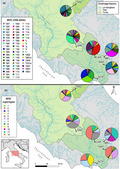

## 1 INTRODUCTION

The major histocompatibility complex (MHC) is an extraordinarily polymorphic, gene‐rich family involved in the immune response of vertebrates. Proteins encoded by classical MHC genes recognize and bind antigens derived from parasites and pathogens initiating the adaptive immune cascade (Kaufman, 2018). The range of antigens that a MHC protein can recognize is determined by the amino acid composition of particular highly variable sites, referred to as antigen‐binding sites, encoded by the exon 2 in classical MHC class II genes.

Positive selection for nonsynonymous mutations, mainly in antigen‐binding sites, and recombination contribute to generating the characteristic high levels of allele and sequence polymorphism at MHC loci (Radwan et al., 2020). Notably, given the adaptive significance and the long‐time persistence of MHC polymorphism, MHC has become a paradigm for investigating pathogen‐mediated balancing selection. Two mutually non‐exclusive forms of balancing selection are commonly postulated: the rare allele advantage (RAA) and the heterozygote advantage (HA) (Radwan et al., 2020; Spurgin & Richardson, 2010).

The RAA, also called negative frequency‐dependent selection, is thought to mainly contribute to maintaining MHC polymorphism by preventing the loss of rare variants. RAA arises from host–parasite arms race (Red Queen dynamics) in which pathogens rapidly adapt to escape recognition by the commonest host MHC alleles, ultimately resulting in cyclical fluctuations of MHC allele frequencies. Computational studies supported the RAA theory (e.g., Borghans et al., 2004) and experimental trials conducted in semi‐natural condition demonstrated that fish carrying novel MHC (functional) variants experienced a reduction of parasite infection (Phillips et al., 2018). As an interesting consequence, the RAA may facilitate the introduction and spread of novel, initially rare alleles into populations via introgression. For instance, Dudek et al. (2019) demonstrated that introgression coupled with balancing selection caused a massive allelic exchange and overall higher MHC variation in a newt hybrid zone.

Under the HA model, MHC heterozygous individuals are predicted to better cope with pathogens because of their ability to recognize a wider range of antigens compared to homozygotes. By extension, heterozygotes carrying genetically divergent MHC alleles should recognize a still broader spectrum of antigens than heterozygotes carrying similar alleles, that is, the divergent allele advantage (DAA) hypothesis (Wakeland et al., 1990). Evidence for HA, such as a significant positive association between MHC heterozygosity and individual fitness‐related traits (e.g., parasite diversity, infection intensity, body condition, survival), has been detected sometimes (e.g., Evans & Neff, 2009; Niskanen et al., 2014; Savage et al., 2019). Nevertheless, other studies detected underdominance (Ilmonen et al., 2007; Pitcher & Neff, 2006) or failed to identify MHC heterozygote excess across populations (e.g., Miller et al., 2001; Monzón‐Argüello et al., 2013, 2014; Talarico, Babik, Marta, Pietrocini, et al., 2019), sometimes due to the lack of statistical power (Garrigan & Hedrick, 2003). Theoretical studies found support for the DAA theory as a mechanism maintaining MHC diversity (Stefan et al., 2019), but a correlation between MHC dissimilarity and proxies of fitness was established occasionally (Eizaguirre et al., 2012; Lenz et al., 2009, 2013; Seifertová et al., 2016). Also, contradictory results emerged when testing in wild populations whether observed MHC genotypes combined more sequence diversity than expected by chance (Gaigher et al., 2018; Monzón‐Argüello et al., 2014; Talarico, Babik, Marta, Pietrocini, et al., 2019).

As mentioned above, contemporary pathogen‐mediated balancing selection can be assessed at the individual level by testing the association between fitness‐related traits and components of MHC diversity, for example, sequence dissimilarity, frequency of variants, zygosity, and number of alleles (Lenz et al., 2009, 2013; Seifertová et al., 2016; Trachtenberg et al., 2003). On the other hand, exploring selection over the history of populations requires comparing levels of diversity and/or genetic structuring between MHC and putative neutral‐evolving loci, such as microsatellites (Garrigan & Hedrick, 2003). Similar levels of structuring at MHC and neutral loci are expected when demographic processes overcome selection in shaping MHC diversity across populations. Conversely, a weaker or stronger MHC structuring compared to that of neutral loci may indicate either uniform balancing selection or fluctuating selection/local adaptation, respectively (Garrigan & Hedrick, 2003; Spurgin & Richardson, 2010). In addition, mixed and/or scale‐dependent patterns are possible (Miller et al., 2001; Talarico, Babik, Marta, Pietrocini, et al., 2019). Therefore, which balancing selection processes underpin MHC diversity is still an open question, and the relative contribution of each process remains unclear, especially in wild populations under natural or semi‐natural condition.

Another innovative perspective concerns the functionality of MHC diversity, with MHC studies increasingly focusing on MHC supertypes. Supertypes are classes of sequences with similar biochemical properties at those amino acid positions determining the specificity of antigen binding (Schwensow et al., 2007; Sette & Sidney, 1998). The supertyping approach has the advantage of accounting for the (actual) functional diversity of MHC variants, which is biologically relevant especially in ecological studies where phenotypic effects of MHC alleles should be examined (Naugler & Liwski, 2008; Trachtenberg et al., 2003). Moreover, supertyping implies reducing the usually high number of MHC variants that often represents a challenge for statistical analyses (Schwensow et al., 2007; Sommer, 2005). For instance, associations between specific supertypes and pathogen resistance, lifetime reproductive success, and survival were found in the wild great tit (Sepil et al., 2012, 2013), and an advantage for rare MHC supertypes in HIV disease progression was detected in human (Trachtenberg et al., 2003). On an evolutionary perspective, Lighten et al. (2017) innovatively proposed a different role of selection on MHC supertypes compared to alleles. According to this hypothesis, balancing selection would maintain functional (supertype) diversity, while positive selection and/or genetic drift would erode allelic diversity, giving rise to a population structuring more pronounced for MHC alleles than supertypes. Nevertheless, Ejsmond et al. (2018) questioned empirical support to this hypothesis, and a successive study failed to confirm such a dual role of selection in the same target species (Herdegen‐Radwan et al., 2020).

Brown trout is one of the most genetically and phenotypically variable species, referred to as *Salmo trutta* complex, and among the most broadly distributed salmonids worldwide (Lobón‐Cerviá & Sanz, 2018). The species complex includes several genetic lineages, which extensively hybridize with hatchery‐reared brown trout (typically domestic lineages of Atlantic origin), released into the wild for restocking purposes for more than one century (reviewed in Lobón‐Cerviá & Sanz, 2018). Various native lineages occur in Italy and inhabit inland freshwaters, mostly upper mountain streams and, occasionally, lowland cold waters. Artificial and/or natural barriers (e.g., dams, waterfalls, fluctuations of water flow) interrupt hydrological connectivity giving rise to genetically structured wild populations with low effective sizes, moreover frequently biased by restocking with domestic trout (Berrebi et al., 2019; Fabiani et al., 2018; Rossi et al., 2019; Splendiani et al., 2019).

Previous studies targeting brown trout revealed high polymorphisms and historical positive selection on the MHC class II gene (the single‐copy MHC‐DAB) and examined multiple aspects of MHC variation and evolution. Campos et al. (2006) characterized MHC‐DAB and neutral variation across native or artificially founded populations in Spain and investigated forces driving MHC evolution in the wild. The effect of genetic drift and successive invasion waves on MHC genetic structure and (functional) diversity was explored in introduced brown trout from the southern hemisphere (Monzón‐Argüello et al., 2014), while Schenekar and Weiss (2017) compared MHC‐DAB diversity and selection strength between native Austrian populations and hatchery stocks. At last, experimental studies assessed the relationship between MHC genotypes/alleles and fitness (reproductive success, resistance against pathogens) to scrutinize MHC‐based mate choice (Forsberg et al., 2007; Jacob et al., 2010).

Here, we characterize putative adaptive (MHC) and neutral (microsatellite loci) variation in genetically unrelated wild populations of brown trout from central Italy. The study area was previously recognized as a hotspot of genetic diversity for native brown trout lineages, although almost all populations were variously subject to stocking with domestic brown trout (Fabiani et al., 2018; Rossi et al., 2019). Such activities may ultimately favor introgression—which refers to the consequence of hybridization between lineages rather than distinct species in this case—and maintenance of novel genetic variation into wild native populations through balancing selection mechanisms, especially at putatively adaptive genes such as MHC. For these reasons, we used brown trout as a suitable model to investigate and disentangle evolutionary forces driving both neutral and adaptive diversity in a context of genetic disturbance caused by human activities. Specifically, the purposes of this study are twofold. Firstly, we aim to assess the relative contribution of selection and nonselective forces, i.e., genetic drift and (human‐mediated) gene flow, to drive putatively adaptive MHC variation in the recent past, by comparing genetic structuring and diversity at MHC and microsatellite loci, while controlling for the extent of admixture between native and domestic lineages. Secondly, we look for signatures of current balancing selection by testing the association between individual body condition (a putative proxy of parasite load) and frequency of MHC variants carried by an individual (i.e., testing for rare allele advantage), MHC zygosity (i.e., testing for heterozygote advantage), and within‐individual MHC dissimilarity (i.e., testing for divergent allele advantage). We also explored associations between specific MHC functional variants (supertypes) and body condition.

## 2 MATERIALS AND METHODS

### 2.1 Sample collection and DNA extraction

**FIGURE 1:**
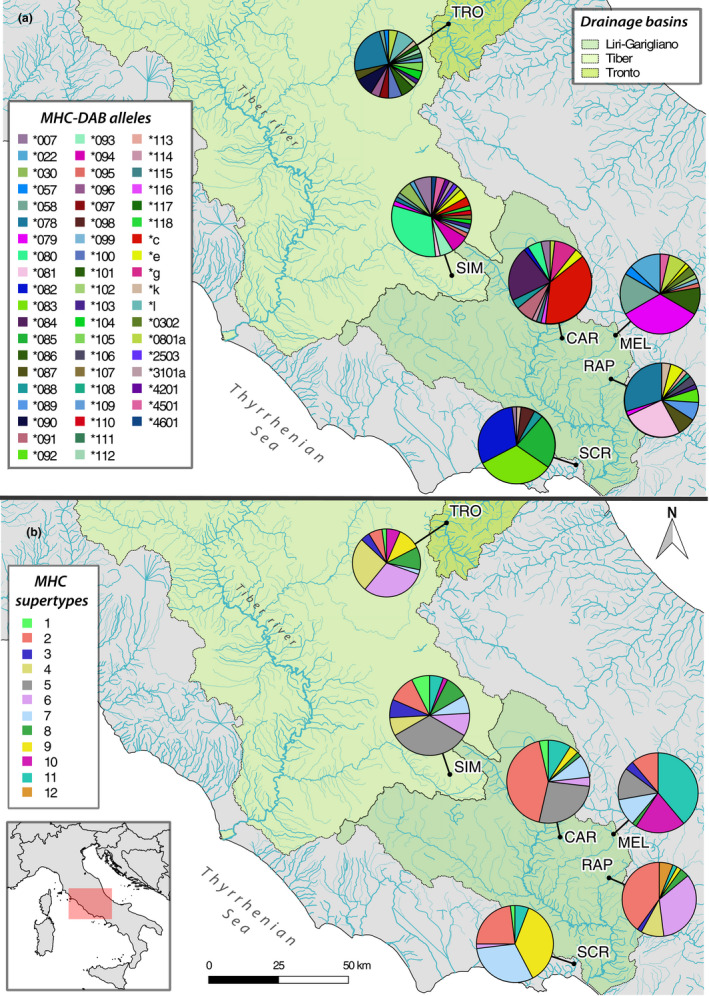
Frequency and geographic distribution of 58 MHC‐DAB alleles (a) and 12 MHC supertypes (b) across six wild brown trout populations from three drainage basins in central Italy. The geographic location of the study area (red rectangle) is displayed in the bottom‐left map. Population abbreviations refer to Table 1; pie charts are proportional to sample sizes

During summer 2019, 156 wild brown trout were sampled by electrofishing from six putatively unrelated populations in central Italy (Latium) (Table 1; Figure 1). For each fish, we collected a fin clip and recorded weight and standard length (i.e., the total length excluding the caudal fin) before releasing in the field. Fin clips were stored in ethanol at −20°C until total DNA extraction, which was carried out following a salt‐extraction protocol (Aljanabi & Martinez, 1997). The “Direzione agricoltura, promozione della filiera e della cultura del cibo, caccia e pesca” and “Direzione Regionale Politiche Ambientali e Ciclo dei Rifiuti” of the Latium Region approved all sampling procedures (authorization no. G10101‐27/7/2019 and Q169/2019).

**TABLE 1:** Genetic variation at the MHC, when considering alleles (MHC‐DAB) or supertypes (MHC‐ST), and 11 microsatellite loci (STR) for six wild brown trout populations from central Italy

Pop	River drainage					LDH (%)		MHC‐DAB					MHC‐ST			STR				
		Elev (m)	Lat° (N)	Lon° (E)	N_ind_	Native	Domestic	N	R	P	Ho	He	N	R	P	N	R	P	Ho	He
CAR	Rio Carpello	293	41.70	13.68	28	85.7	14.3	13	10.87	6	0.86	0.81	8	7.30	0	6.91	5.98	1.36	0.47	0.51
MEL	Melfa	1,159	41.70	13.91	27	9.3	90.7	12	10.37	2	0.81	0.84	7	6.57	0	6.09	5.72	0.45	0.69	0.73
RAP	Rapido	154	41.56	13.88	25	54.0	46.0	12	10.63	5	0.88	0.83	8	7.03	1	7.45	6.93	0.82	0.78	0.74
SCR	Rio Santa Croce	37	41.29	13.71	26	94.2	5.8	7	6.26	3	0.65	0.75	6	5.36	0	4.91	4.50	1.00	0.42	0.41
SIM	Simbrivio	701	41.92	13.22	27	61.1	38.9	25	19.04	10	0.67*	0.89	10	9.60	0	10.64	9.29	2.55	0.72	0.74
TRO	Tronto	693	42.73	13.26	23	54.3	45.7	20	17.39	11	0.91	0.92	9	8.42	0	9.82	9.02	1.64	0.76	0.77

Note

For each population (Pop): the river drainage; elevation (Elev); geographic coordinates (datum WGS84); the number of genotyped individuals (N_ind_); the frequency of the native *100 and the domestic *90 lactate dehydrogenase (LDH) alleles; the number of alleles/supertypes (N) and private alleles/supertypes (P); the allelic/supertype richness (R); and the observed (Ho) and expected (He) heterozygosity. For the MHC‐DAB, significant (*p* < .05) deviations from Hardy–Weinberg expectations are indicated with an asterisk.

### 2.2 Amplification and genotyping of MHC, STR loci, and the LDH gene

To assess putative adaptive variation, we PCR‐amplified a 254–257 bp fragment of the highly polymorphic exon 2 of the MHC class II DAB gene (hereafter MHC‐DAB). Individually tagged PCR amplicons were pooled in batches and subsequently sequenced on an Illumina MiSeq platform (Illumina—San Diego, CA, USA). Preprocessing of output reads and MHC genotyping were performed with AmpliSAT tools (Sebastian et al., 2016). Both laboratory procedures and bioinformatics are extensively described in Appendix S1.

Individuals were genotyped at 11 polymorphic microsatellite loci (hereafter STR) to estimate neutral diversity (details on amplification and genotyping procedures are given in Appendix S2). Additionally, we followed the RFLP‐based method described in McMeel et al. (2001) to obtain individual profiles of the lactate dehydrogenase (LDH) C1 gene, which is routinely used to assess the admixture between native and domestic lineages: the LDH*90 allele is fixed within European hatchery stocks of domestic brown trout, while the *100 allele naturally occurs in wild, Mediterranean‐native brown trout populations.

### 2.3 MHC characterization: Historical positive selection, recombination, and supertyping

Novel MHC‐DAB alleles were named according to Klein et al. (1990). Sequences were manually aligned in MEGA7 (Kumar et al., 2016). To effectively visualize the complex genealogies among MHC variants, we built a neighbor‐net network using the Kimura two‐parameter distance in SplitsTree5 (Huson & Bryant, 2006). The analysis of relationships included newly identified alleles and 98 unique MHC‐DAB sequences of *Salmo trutta* deposited in GenBank which allowed a full‐length alignment: DQ257387‐DQ257410 (Campos et al., 2006); DQ491027–DQ491060 (Forsberg et al., 2007); HM596877‐HM596889 (Jacob et al., 2010); and KT003641‐KT003667 (Schenekar & Weiss, 2017).

Two approaches were adopted to look for signatures of historical positive selection on MHC‐DAB sequences. Firstly, the one‐tailed *Z* test performed in MEGA7 was used to test for *ω* > 1 (i.e., the ratio between nonsynonymous and synonymous substitution rates, according to the Nei–Gojobori method of pairwise comparison with the Jukes–Cantor correction; Nei & Gojobori, 1986) in three alignment partitions: all sites; only positively selected sites (PSSs) detected in brown trout (see below); and only non‐PSSs. Secondly, we choose the best‐fitting model, based on the minimum Akaike information criterion (AIC) value, among three codon‐based evolution models as performed in PAML v4.8 (Yang, 2007): M0 assuming a single ω value for all codons; M7 considering purifying selection and neutrality (*ω* ≤ 1); and M8 allowing evolution by positive selection (*ω* > 1) in a proportion of sites. We run the Single BreakPoint recombination algorithm (SBP; Kosakovsky Pond et al., 2006) on the Datamonkey server (Delport et al., 2010) to detect putative recombination events.

To account for functional MHC diversity, MHC alleles were grouped into theoretical supertypes following a widely adopted statistical approach (Doytchinova & Flower, 2005), which accounts for immunological (Sidney et al., 1996) and laboratory (Sandberg et al., 1998) concepts. To this end, we performed six tests to identify positively selected sites (PSSs), namely putative amino acid sites involved in peptide binding, and conservatively retained only PSSs detected by at least two tests among the Bayes empirical Bayes (BEB; Yang et al., 2005); the fast unconstrained Bayesian approximation (FUBAR; Murrell et al., 2013), the mixed‐effects model of evolution (MEME; Murrell et al., 2012), the single‐likelihood ancestor counting (SLAC), the fixed effects likelihood (FEL), and the random‐effects likelihood (REL) analyses (Kosakovsky Pond & Frost, 2005). The analyses involved novel alleles plus 98 unique sequences retrieved from GenBank (see above) to improve detection accuracy and robustness of PSSs. Then, detected PSSs were extracted from sequences and each PSS was characterized according to five physicochemical descriptors as in Sandberg et al. (1998). Supertypes were defined using the K‐means clustering algorithm implemented in the adegenet R‐package (Jombart, 2008) and the most probable number of supertypes was that returning the lowest value of the Bayesian information criterion (BIC). Finally, all alleles within each cluster were collapsed into a single MHC supertype. For downstream analyses, we used MHC genotypes resulting from either alleles or supertypes (referred to as MHC‐DAB and MHC‐ST “loci,” respectively).

### 2.4 Population structure and diversity at MHC and STR

As a preliminary step, we performed the STR‐based Bayesian clustering analysis in STRUCTURE v.2.3.4 (Pritchard et al., 2000) to ensure that studied populations were genetically unrelated. The STRUCTURE clustering confirmed our a priori assumption and, consequently, populations were treated separately for most of the downstream analyses. Methods and results concerning the STRUCTURE analysis are reported in Appendix S3.

In FreeNA (Chapuis & Estoup, 2007), we explored the frequency of null alleles for each locus and population according to the Dempster's EM algorithm. For both MHC‐DAB and STR, departures from Hardy–Weinberg equilibrium and linkage disequilibrium were tested in GenePop v.4.7 (Rousset, 2008) within each population, adjusting *p*‐values for multiple testing (Holm–Bonferroni correction). To estimate population genetic variability, we calculated the number of alleles, the number of private alleles, and the observed and expected heterozygosity in GenAlEx 6.5 (Peakall & Smouse, 2012). Additionally, to compare diversity among populations with different sample sizes (see Table 1), we calculated allelic richness by rarefaction (minimum sample size = 23) in the PopGenReport R‐package (Adamack & Gruber, 2014). All indices were computed for STR, MHC‐DAB, and MHC‐ST separately.

We fitted linear models (lm function in R; R Core Team, 2020) to evaluate the contribution of neutral variation (*STR richness*) and admixture (*LDH‐admixture*) to determine MHC diversity (*MHC‐DAB richness* and *MHC‐ST richness*) across populations. The fitted models followed the general formula:

MHCrichness∼STRrichness+LDHadmixture

Note that *LDH‐admixture* indicates the frequency of the less common LDH allele (typically the domestic *90 allele) within a population. As LDH is a biallelic locus, a population is completely admixed between native and domestic lineages when *LDH‐admixture* is 50% (i.e., 50% of *100 and 50% of *90). Conversely, low levels of *LDH‐admixture* indicate a “pure” population, either native or domestic. To circumvent the collinearity among *STR richness* and *LDH‐admixture* (Spearman *r*_s_ = 0.77, *p* = .10), we used a sequential regression approach (Dormann et al., 2013; Graham, 2003). Here, *LDH‐admixture* was regressed against *STR richness* and the residuals of this model were used to represent the portion of variation in *LDH‐admixture* not accounted for by *STR richness*. Both *LDH‐admixture* and *STR richness* were scaled to zero mean and unit variance before modeling to allow a full comparability of regression coefficients.

In GenAlEx, we calculated overall and population‐pairwise genetic differentiation (i.e., the Nei's G_ST_ for highly polymorphic markers with correction for a small number of populations) for STR loci, the MHC‐DAB, and the MHC‐ST. Also, since collapsing MHC alleles into supertypes may ultimately cause biased patterns of population structuring, we tested whether the observed supertype‐based population G_ST_ was significantly different than expected by chance. Following Lighten et al. (2017), we randomly assigned 10,000 times MHC alleles to supertypes, calculating each time per‐population G_ST_. Observed G_ST_ values significantly higher or lower than the null distribution of G_ST_ may reflect a biological phenomenon (i.e., strong diversifying or balancing selection, respectively), while nonsignificant differences may reflect a bioinformatic artifact (Herdegen‐Radwan et al., 2020; Lighten et al., 2017), thus suggesting caution in interpreting the observed degree of functional population structuring.

Aiming at identifying signatures of selection on the MHC, we performed multiple analyses comparing genetic structure resulting from presumably neutral (STR) and adaptive (MHC) loci. Firstly, we tested for the correlation between G_ST_ matrix pairs (MHC‐DAB vs. STR and MHC‐ST vs. STR) using Mantel tests (9,999 permutations) in PAST v3.26 (Hammer et al., 2001). Secondly, we carried out two outlier analyses of diversifying/balancing selection in BayeScan 2.1 (Foll & Gaggiotti, 2008) and Arlequin v3.5 (Excoffier & Lischer, 2010). The former uses Bayesian inference to estimate, for each locus, a posterior probability for the model including selection. The latter computes per‐locus confidence intervals of *F*_ST_ values expected under neutrality as a function of heterozygosity with a coalescent approach. Loci in the tails of the simulated distribution (outliers) are regarded as under balancing (lower structure than neutral expectations) or diversifying (stronger structure than neutral expectations) selection. Both outlier analyses were run with default settings and including STR loci and the MHC‐DAB. Thirdly, we investigated whether the (effective) gene flow from hatcheries to wild populations was more or less pronounced for MHC compared to STR. To this aim, we grouped massively stocked populations (those with >30% frequency of the LDH*90 allele) and relatively pure native populations (LDH*90 frequency <15%) and performed hierarchical analyses of molecular variance (AMOVA, 10,000 permutations in Arlequin v.3.5; Excoffier & Lischer, 2010) for STR loci, MHC‐DAB, and MHC‐ST separately. We expected levels of variation explaining differences between native and massively stocked population groups to be (a) similar for MHC and STR in case of comparable rates of effective gene flow (introgression from domestic lineages comparable for MHC and neutral genes); (b) lower for MHC than STR in case of increased effective gene flow for MHC (selection promotes the introgression of MHC genes); and (c) greater for MHC than STR, in case of a reduced effective gene flow for MHC (selection hinders the introgression of MHC genes).

### 2.5 Relationship between body condition and MHC

We used mixed‐effects linear models (performed in the R‐package lme4; Bates et al., 2015) to look for signatures of current balancing selection acting on MHC in the form of heterozygote advantage, divergent allele advantage, and rare allele advantage. Specifically, we assessed the effect of MHC zygosity and frequency of MHC variants (model 1) and MHC dissimilarity (model 2) on *body condition*, while controlling for the genetic background (*STR heterozygosity* and *LDH type*) and origin (*population*) of individuals—the latter was included in the models as a random intercept to account for possible ecological differences (e.g., food availability) across populations that may affect *body condition*. Model formulas were as follows:

Model1:Bodycondition∼MHCfrequencyindex+MHCzygosity+STRheterozygosity+LDHtype+population

Model2:Bodycondition∼MHCdissimilarity+STRheterozygosity+LDHtype+population

where:

- *Body condition* refers to the Fulton's condition factor (computed as 100 · weight/standard length^3^), a commonly used index of general condition which assumes that heavier fish of a given length are in better condition.
- *MHC frequency index* is simply the sum of the within‐population frequency of both alleles (MHC‐DAB) or supertypes (MHC‐ST) carried by an individual. For homozygotes, the index corresponds to the doubled within‐population frequency of the single MHC variant carried by an individual. Thus individuals carrying locally rare MHC variants show a low *MHC frequency index* value, conversely to those carrying locally common variants.
- *MHC zygosity* is a two‐level factor coding heterozygotes and homozygotes at the MHC‐DAB and, separately, at the MHC‐ST. Because *MHC frequency index* and *MHC zygosity* are intrinsically related (Spearman *r* = −0.44 and −0.38 for MHC alleles and supertypes, respectively, with *p* < .001)—rare variants mostly occur in heterozygotes, while abundant ones occur more frequently in homozygotes—we adopted the previously described sequential regression approach (Dormann et al., 2013; Graham, 2003). *MHC zygosity* was regressed against *MHC frequency index* using a generalized linear model with binomial error distribution, and the residuals from this model were used to represent the portion of variation in *MHC zygosity* not accounted for by *MHC frequency index*.
- *MHC dissimilarity* is the pairwise distance between MHC‐DAB sequences carried by an individual, measured in MEGA7 as the nucleotide Kimura two‐parameter distance (correcting for multiple hits) and the Poisson‐corrected amino acid distance (adjusting for multiple substitutions). Note that *MHC dissimilarity* is set to 0 for MHC‐DAB homozygous individuals.
- *STR heterozygosity* is the proportion of heterozygous STR loci, as performed in GenAlEx, approximating the genome‐wide neutral diversity.
- *LDH type* is a three‐level factor indicating the genotype at the LDH locus, namely “native” = 100/100, “hybrid” = 90/100, and “domestic” = 90/90.

Finally, we examined the influence of specific MHC variants on body condition following the approach described in Sepil et al. (2012). In brief, we compared the baseline model (i.e., the previously defined model 1, which accounts for the effects of frequency of MHC variants carried by an individual, MHC zygosity status, genetic background, and population origin) to models including the baseline and the effect of single specific MHC variants (coded as presence/absence). Then, we ranked models by increasing AICc (AIC corrected for small sample size), namely from the best to the worst, and inspected the significance of beta coefficients (effect sizes) of models fitting the data substantially better than the baseline model, that is, those with an AICc at least 2 points lower than the baseline (Burnham & Anderson, 2002).

## 3 RESULTS

### 3.1 MHC characterization

**FIGURE 2:**
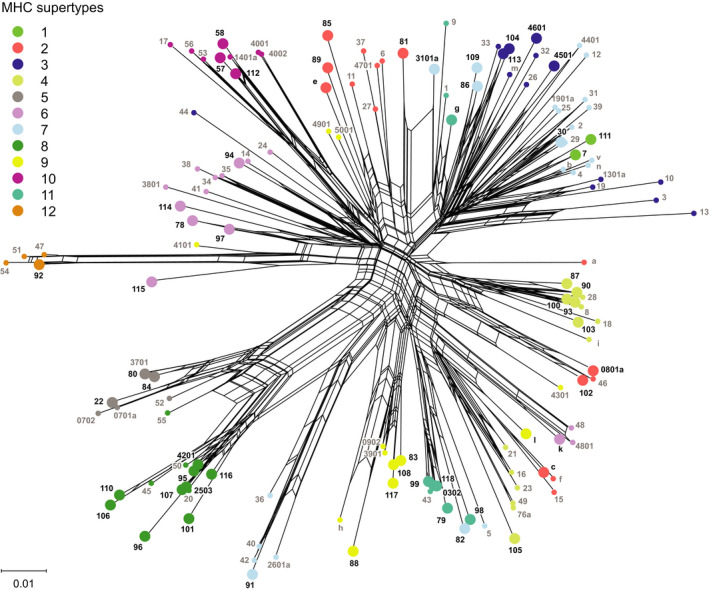
Neighbor‐net network (Kimura two‐parameter distance) based on overall 139 MHC‐DAB alleles of brown trout, 58 of which found in populations examined in this study (indicated by black labels and larger circles). Colors, consistent with those in Figure 1b, identify alleles clustered into identical supertypes according to physicochemical properties of the 28 positively selected amino acid sites (PSSs) shown in Appendix S4

We sequenced a fragment of MHC‐DAB exon 2 in 156 wild brown trout from six populations. After bioinformatic processing of raw data, the mean individual coverage was 6,833.8 reads (±4,382.0 *SE*). All individuals were successfully genotyped and showed 1–2 variants, which reads represented on average the 73.1% (±15.3% *SE*) of individual coverage. This confirmed a single MHC‐DAB locus amplified with no individual copy‐number variation. In total, we obtained 58 MHC‐DAB alleles of 254 or 257 bases, each translating into a unique amino acid sequence (among them, 41 were novel; Appendices S4 and S5), and identified 28 PSSs (Appendix S4). Based on the physicochemical properties of the assumed PSSs, novel MHC‐DAB alleles and sequences retrieved from GenBank were clustered into 12 supertypes (Figure 2; Appendix S5). The reconstruction of allele genealogies showed a generally high sequence divergence among alleles, although several clusters well matched the supertype grouping (i.e., supertype‐5, supertype‐8, supertype‐10, and supertype‐12; Figure 2). The MHC‐DAB gene showed clear evidence for historical positive selection: the M8 model indicating positive selection was strongly supported (ΔAIC = 277.5; Appendix S6), and the excess of nonsynonymous substitutions was statistically significant both considering all 85 amino acid positions (*Z* test in MEGA: *Z* = 4.156, *p* < .001) and 28 PSSs (*Z* = 6.527, *p* < .001), but not the 57 non‐PSSs (*Z* = 0.077, *p* = .47). The SBP analysis revealed a putative recombination breakpoint at the nucleotide site 174 (corresponding to amino acid site 58; Appendix S4).

### 3.2 Genetic variation in studied populations

The frequency of null alleles was negligible for all loci and populations, being barely >10% in MEL and SCR at the ONEPHI2 locus only (Appendix S7). A significant heterozygote deficiency was found in RAP at the STR locus SSOSL417 and in SIM at the MHC‐DAB (Appendix S8). Linkage disequilibrium was statistically significant (*p* < .05) in only 10 out of 396 pairwise comparisons, nine of which occurred in RAP, hence indicating that there was no physical linkage between any loci. Consequently, we retained all STR loci as the reference for neutral variation for subsequent analyses.

A summary of population genetic variation is shown in Table 1. MHC‐DAB allelic richness ranged between 6.3 and 19.0. Private alleles per population were on average 40.4% (± 11.7% *SE*). In each population, one to four alleles had a higher frequency (>10%), while the others occurred at lower frequencies (Figure 1a; Appendix S5). We found 6–10 MHC supertypes per population; supertype‐12 occurred only in RAP, but others were shared among at least three populations (Figure 1b; Appendix S5). The distribution of alleles and supertypes did not reveal any evident geographic pattern (Figure 1), for example, an obvious differentiation among drainage basins or among rivers draining into Adriatic versus Tyrrhenian Sea. The domestic LDH*90 allele was found in all examined populations varying considerably in frequency (Table 1). Particularly, the *90 allele prevailed in MEL, likely due to the impact of massive stocking, while the LDH composition in SCR and CAR revealed the nearly pure native origin of these populations.

### 3.3 Comparison between STR and MHC population diversity and structuring

Linear models revealed a strong, positive relationship between neutral diversity (*STR richness*) and MHC diversity (both *MHC‐DAB richness* and *MHC‐ST richness*) (Table 2). *LDH‐admixture* was a significant predictor of *MHC‐DAB* richness showing an overall positive correlation, but its contribution to *MHC‐ST richness* was statistically nonsignificant.

**TABLE 2:** Results of linear models with MHC diversity (*MHC‐DAB richness*) and functional diversity (*MHC‐ST richness*) as the dependent variable, and microsatellite diversity (*STR richness*) and admixture between native and domestic brown trout lineages (*LDH‐admixture*) as predictors

	MHC‐DAB richness	MHC‐ST richness
*Intercept*	12.43*** (0.10)	7.38*** (0.19)
*STR richness*	4.71*** (0.11)	1.40** (0.21)
*LDH‐admixture*	−1.02** (0.11)	−0.29 (0.21)
Adjusted *R* ^2^	0.997***	0.903*
No. of observations	6	6

Note

The estimates of intercept and coefficients for predictors, the coefficient of determination (adjusted *R* ^2^), and the number of observations (no. of observations) are given for the two models. Note that, since we applied a sequential regression approach, the coefficient estimates for *LDH‐admixture* indicate the relative effect that admixture has once accounted for the positive effect of *STR richness*—thus negative values indicate a relative effect, while the overall effect (i.e., without accounting for *STR richness*) is positive. Standard errors are reported in parentheses; statistical significance (*p < .05; **p < .01; and ***p < .001) is referred to *t* tests and *F* test for intercept/predictors and *R* ^2^, respectively.

Differentiation (G_ST_) was statistically significant for all markers and population comparisons (Appendix S9). Overall G_ST_ among populations was 0.15 for MHC‐DAB, 0.14 for MHC‐ST, and 0.22 for STR loci (95% confidence interval: 0.18–0.27). The observed supertype‐based G_ST_ was not significantly different from those obtained with randomizations (i.e., random lumping of alleles into supertypes) in each population (Appendix S10). Neutral (STR‐based) population differentiation significantly and positively correlated with MHC‐DAB‐based differentiation (Mantel *r* = 0.87, *p* = .002), but not with genetic structuring of MHC‐ST (Mantel *r* = 0.30, *p* = .173). BayeScan did not detect outliers with a false discovery rate for multiple testing (*q*‐value) <0.05, while three STR loci (SSA408UOS, SSA85, and SSA413NVH) were putative outliers in the Arlequin analysis with *p* < .05. MHC‐DAB was never identified as an outlier. After grouping massively stocked (MEL, RAP, SIM, and TRO) and relatively pure (CAR and SCR) populations, the hierarchical AMOVAs indicated that the amount of differentiation among groups was larger for STR than MHC even though marginally supported (STR: *p* = .063; MHC‐DAB: *p* = .069) or not supported (MHC‐ST: *p* = .332) (Table 3). Variation within populations accounted for the largest fraction of variance (>74%), as expected for highly polymorphic markers.

**TABLE 3:** Results of hierarchical analysis of molecular variance (AMOVA) for MHC alleles (MHC‐DAB), MHC‐supertypes (MHC‐ST) and 11 microsatellite loci (STR)

Source of variation	STR	MHC‐DAB	MHC‐ST
Among groups *native* (*CAR* + *SCR*) vs. *stocked* (*MEL* *RAP* + + *SIM* + *TRO*)	9.3^†^	2.3^†^	0.8
Among populations within groups	16.3***	13.7***	13.7***
Within populations	74.3***	84.0***	85.5***

Note

The amount of variation (%) explained by differences between native and massively stocked populations, among populations within groups and within populations are given along with the significance level (^†^ *p* < .1; *p < .05; **p < .01; and ***p < .001).

### 3.4 Relationship between body condition and MHC

The relationship between multiple components of MHC diversity and body condition was tested through mixed‐effect models (Table 4), which showed that (a) the within‐population frequency of MHC alleles and supertypes carried by an individual was negatively associated with its body condition (model 1, *p* < .01), while zygosity was unrelated with *body condition* for either MHC‐DAB and MHC‐ST (model 1, *p* = .13 and 0.18, respectively); and (b) nucleotide and amino acid dissimilarity between MHC sequences carried by an individual was not a significant predictor of *body condition* (model 2, *p* = .10 and 0.11, respectively). The effect of other genetic predictors included in the models (i.e., *LDH type* and *STR heterozygosity*) was statistically nonsignificant (Table 4).

**TABLE 4:** Results of linear mixed‐effects models testing the relationship between *body condition* (Fulton's condition factor) and MHC diversity in 156 brown trout, while controlling for genome‐wide neutral diversity (*STR heterozygosity*, the proportion of heterozygous STR loci), genotype at the LDH locus (*LDH type*), and population origin

Model	Variable	Estimate (SE)	*df*	*t*	*p*	*R* ^2^m	*R* ^2^c
1_DAB_	***Intercept***	**1.52 (0.07)**	**83.0**	**21.4**	**<.001**	0.11	0.22
	***MHC‐DAB frequency index***	**−0.15 (0.06)**	**148.9**	**−2.4**	.**02**		
	*MHC‐DAB zygosity*	0.02 (0.01)	149.4	1.5	.13		
	*STR heterozygosity*	0.12 (0.08)	96.3	1.4	.16		
	*LDH type*—hybrid (90/100)	−0.04 (0.03)	136.1	−1.1	.28		
	*LDH type*—native (100/100)	−0.01 (0.04)	103.7	−0.4	.70		
1_ST_	***Intercept***	**1.53 (0.07)**	**72.6**	**21.3**	**<.001**	0.11	0.23
	***MHC‐ST frequency index***	**−0.16 (0.06)**	**148.1**	**−2.6**	.**01**		
	*MHC‐ST zygosity*	0.02 (0.06)	149.5	1.4	.18		
	*STR heterozygosity*	0.13 (0.08)	90.2	1.6	.11		
	*LDH type*—hybrid (90/100)	−0.03 (0.03)	140.8	−0.9	.34		
	*LDH type*—native (100/100)	−0.01 (0.04)	110.5	−0.28	.78		
2_NU_	***Intercept***	**1.41 (0.07)**	**57.5**	**21.4**	**<.001**	0.07	0.17
	*MHC dissimilarity (nucleotide)*	0.42 (0.25)	149.2	1.6	.10		
	*STR heterozygosity*	0.16 (0.08)	72.7	2.0	.05		
	*LDH type—hybrid (90/100)*	−0.05 (0.03)	134.8	−1.3	.19		
	*LDH type—native (100/100)*	−0.02 (0.04)	95.4	−0.4	.68		
2_AA_	***Intercept***	**1.41 (0.07)**	**56.8**	**21.5**	**<.001**	0.07	0.17
	*MHC dissimilarity (amino acid)*	0.19 (0.12)	148.6	1.6	.11		
	*STR heterozygosity*	0.16 (0.08)	73.1	1.9	.06		
	*LDH type—hybrid (90/100)*	−0.04 (0.03)	135.7	−1.3	.20		
	*LDH type—native (100/100)*	−0.01 (0.04)	95.6	−0.4	.69		

Note

MHC diversity is measured as: within‐population frequency of alleles (*MHC‐DAB frequency index*) and supertypes (*MHC‐ST frequency index*) carried by an individual and its zygosity (based on alleles, *MHC‐DAB zygosity*, and supertypes, *MHC‐ST zygosity*) in model 1; *MHC dissimilarity* based on nucleotide (Kimura two‐parameter) and amino acid (Poisson‐corrected) distances in model 2. Estimates for each model version are provided along with standard errors in parentheses (*SE*), degree of freedom (*df*), *t*‐statistics (*t*), associated *p*‐values (*p*), marginal *R*‐squared (*R* ^2^m, i.e., the variance explained by only fixed effects), and conditional *R*‐squared (*R* ^2^c, i.e., the variance explained by both fixed and random effects). Reference level for factor *LDH type* is the “domestic” genotype 90/90. Variables with a significant effect (*p* < .05) are in bold.

Mixed‐effect models were also used to explore the relationship between specific MHC supertypes and body condition—this was not doable for MHC alleles, as only three alleles occurred with a reasonable sample size (>15 individuals). Among the five models with AICc lower than the baseline model, only those including supertype‐7, supertype‐5, and supertype‐11 were substantially better supported (ΔAIC < −2; Table 5). Beta coefficients of the three top models were significant, with a positive effect (i.e., better body condition) for individuals carrying supertype‐7, and a negative effect (i.e., worse body condition) for those carrying supertype‐5 and supertype‐11.

**TABLE 5:** Results of modeling the effect of specific MHC supertypes on the body condition of 156 Mediterranean brown trout

model	*df*	log(L)	AICc	ΔAICc	beta	*N*
baseline + supertype−5	9	88.47	−157.71	−3.47	**−0.07**	32
baseline + supertype−7	9	88.22	−157.20	−2.96	**0.07**	28
baseline + supertype−11	9	88.16	−157.09	−2.85	**−0.07**	28
baseline + supertype−1	9	87.46	−155.69	−1.45	−0.11	7
baseline + supertype−9	9	86.97	−154.71	−0.47	0.06	23
baseline	8	85.61	−154.24	0	–	–
baseline + supertype−6	9	86.01	−152.78	1.46	−0.03	35
baseline + supertype−10	9	86.00	−152.76	1.48	0.04	15
baseline + supertype−8	9	85.99	−152.75	1.49	0.04	12
baseline + supertype−2	9	85.83	−152.43	1.81	0.02	58
baseline + supertype−4	9	85.73	−152.24	2.00	0.02	21
baseline + supertype−12	9	85.62	−152.01	2.23	0.01	3
baseline + supertype−3	9	85.62	−152.00	2.24	0.01	9

Note

For each model: *df*, the degrees of freedom; log(L), the logarithm of the likelihood; AICc, the Akaike information criterion corrected for small sample size; ΔAICc, the difference in AICc compared to the baseline model (corresponding to model 1_ST_ in Table 4); beta, the effect size of the specific supertype (values with *p* < .05 are given in bold); and *N*, the number of individuals carrying the specific supertype. Models are ranked by increasing AICc.

## 4 DISCUSSION

We characterized adaptive (MHC) and neutral (STR) variation in six brown trout populations from central Italy, which are/were exposed to introgression from domestic lineages. Our ultimate goal was to assess the contribution of selection versus neutral forces to drive MHC variation and testing for the occurrence of balancing selection in its forms. We also evaluated the impact of anthropogenic introgression on MHC diversity.

### 4.1 Characterization of MHC polymorphism and population variability

The analysis of MHC‐DAB polymorphism confirmed typical features of expressed MHC genes, most of which were previously reported in studies targeting brown trout (Campos et al., 2006; Monzón‐Argüello et al., 2014; Schenekar & Weiss, 2017): high levels of sequence variability (e.g., 48.2% of polymorphic amino acid sites), lack of stop codons, and the strong signature of historical positive selection for amino acid replacement, especially in some putative antigen‐binding sites. Identified PSSs were consistent with PSS detected in previous studies focusing on brown trout (Monzón‐Argüello et al., 2014; Schenekar & Weiss, 2017) and significantly matched with corresponding human MHC antigen‐binding sites (*p* < .001, Fisher exact test) described in Brown et al. (1993), hence supporting their robustness. Multiple circular patterns (splits) connecting nodes in the network of MHC‐DAB alleles (Figure 2) indicate, at least in part, reticulate evolution by micro‐recombination (Klitz et al., 2012)—we indeed detected recombination in our sequences—even if they may also imply conflicting phylogenetic signals (Makarenkov et al., 2006). Therefore, positive selection and, to a lesser degree, recombination have generated brown trout MHC‐DAB sequence diversity over the evolutionary timescale. Distinguishing between domestic (Atlantic) and Mediterranean‐native alleles was not possible according to genealogies (Figure 2). Moreover, the retention of MHC polymorphism predating speciation is frequently reported (Těšický & Vinkler, 2015), thus MHC allele sharing among recently diverged brown trout lineages (300,000–1,200,000 years ago; Lobón‐Cerviá & Sanz, 2018) is expected.

To our knowledge, this is the first study reporting MHC variation of Mediterranean brown trout in Italy. We found 58 MHC‐DAB alleles, ranging from 7 to 25 within investigated populations (*N* = 23–28 individuals). The MHC allelic diversity is medium‐to‐higher compared to previous studies: 1–13 alleles (overall 24) were found in 9 native or artificially founded populations (20 individuals each) from the Sella river drainage in Spain (Campos et al., 2006); 9 introduced populations (*N* = 15–23 individuals) from Chile and Falkland Islands exhibited 4–21 alleles each (overall 40; Monzón‐Argüello et al., 2014); 4–9 alleles were detected in three native Austrian populations (*N* = 22–29 individuals), while 10–18 alleles were found in three hatchery stocks (*N* = 20–31 individuals) of Atlantic origin (overall 37 MHC alleles; Schenekar & Weiss, 2017). It is currently unclear which mechanisms may explain the relatively high MHC diversity of Italian populations. Historical processes have substantially affected the geographic distribution of MHC diversity in some taxa, resulting in similar spatial patterns of MHC and neutral diversity (e.g., Cortázar‐Chinarro et al., 2017; Talarico et al., 2019). Therefore, it is possible that Italy—a well‐known hot spot of brown trout genetic diversity (Lobón‐Cerviá & Sanz, 2018)—and the study area in particular (Rossi et al., 2019), may harbor remarkably high levels of diversity even for MHC genes. However, we cannot entirely rule out that high levels of MHC diversity revealed in our study may be partially due to the increased accuracy of the high‐throughput sequencing to detect rare and/or poorly amplified MHC variants, compared to traditional approaches adopted in previous studies (Campos et al., 2006; Monzón‐Argüello et al., 2014; Schenekar & Weiss, 2017).

### 4.2 Neutral evolutionary forces mostly shaped MHC population structure and variation

We found that the pattern of MHC‐DAB variability agreed with neutral expectations: STR population diversity strongly correlated with MHC diversity as expressed by both alleles and functional supertypes, despite the limited number of observations (Table 2); MHC‐DAB population structure substantially matched that of STR loci. Both these pieces of evidence indicate the importance of neutral processes in driving spatial variation of MHC (Garrigan & Hedrick, 2003; Spurgin & Richardson, 2010). Among nonselective forces, we may argue that genetic drift and population demographic history have played a major role to determine the observed differentiation and diversity at the MHC, explaining, at least in part, the among‐population differences in MHC allele frequencies and the occurrence of private alleles. Conversely, migration provided a minor contribution since little gene flow was observed among the examined populations at the investigated spatial scale (results of the STRUCTURE analysis).

The comparative analyses of population structure at MHC‐DAB and neutral loci (outlier analyses, Mantel test) failed to detect strong signals of (pathogen‐mediated) balancing or diversifying selection on the MHC‐DAB, possibly indicating their weak effect in investigated brown trout populations in the recent past (Garrigan & Hedrick, 2003)—but see the discussion section below for balancing selection on the current timescale. Particularly, we did not find signatures of MHC local adaptation, namely a stronger genetic structure for the MHC compared to neutral expectations (STR), which is quite unexpected since pathogens are likely to differ among ecologically heterogeneous biotopes as our study locations (see Table 1, e.g., variation in elevation, but note that pathogen composition information is not available for examined populations).

The analysis of functional structuring did not provide evidence for selection either: randomizations showed that the observed supertype‐based population structure was not substantially different from random expectations; therefore, the lack of correlation between population differentiation at MHC‐ST and STR should be interpreted as an artifact rather than a biological phenomenon. These results also imply that supertypes and MHC alleles “behave” similarly, hence not supporting selection acting differently for MHC alleles and functional supertypes as hypothesized by Lighten et al. (2017).

Despite a relatively large amount of studies, the relative contribution of evolutionary mechanisms to drive the spatial pattern of MHC diversity is still controversial in salmonids. The relevance of neutral forces was demonstrated in various studies, including the present one, focused on both native and artificially founded salmonids populations (Campos et al., 2006; Landry & Bernatchez, 2001; Monzón‐Argüello et al., 2013). Nevertheless, there is no consensus on which forms of selection act on MHC apart from widespread evidence for historical positive selection on PSSs (Landry & Bernatchez, 2001; Miller et al., 2001; Monzón‐Argüello et al., 2013, 2014; Schenekar & Weiss, 2017), possibly as a consequence of their ability to colonize a wide spectrum of habitats. For instance, Landry and Bernatchez (2001) and Hansen et al. (2007), reported signatures for diversifying selection (a more pronounced population structure for MHC than STR) in Atlantic salmon and brown trout, respectively. Directional selection was occasionally invocated to explain a reduction of MHC diversity across generations of rainbow trout outside the native range (Monzón‐Argüello et al., 2013) or a higher population differentiation compared to that revealed by neutral‐evolving markers in wild brown trout (Schenekar & Weiss, 2017). Lastly, Miller et al. (2001) demonstrated spatial heterogeneity in selection intensities and forms, detecting sockeye salmon populations within the same river drainage experiencing balancing or directional selection.

### 4.3 Current balancing selection on MHC: The signature of RAA rather than DAA and HA

We also investigated the occurrence of balancing selection in its various forms on a recent timescale, by testing the association between multiple measures of MHC diversity and fish body condition. This latter is a measure of the general condition of a fish (fitness‐related trait), and it was also found to be negatively related to parasite load in freshwater fish (Kalbe et al., 2012; Seifertová et al., 2016). Thus, it was assumed here as a simple, noninvasive proxy of the parasite load of trout.

After controlling for genetic background (i.e., *STR heterozygosity* and *LDH type*), we found the individuals carrying locally rare MHC alleles/supertypes being in better body condition, irrespective of the *MHC zygosity* or the dissimilarity between MHC variants carried (Table 4). This suggests a selective advantage related to the within‐population (low) frequency of MHC variants, indirectly supporting parasite‐driven balancing selection in the form of RAA. Conversely, we found no evidence for HA from individuals (MHC heterozygous were not associated with better body condition; model 1, Table 4) or populations (no significant heterozygote excess), nor support for DAA (body condition was not correlated with within‐individual MHC dissimilarity; model 2, Table 4). Therefore, we may deduce that RAA is the leading balancing selection process maintaining the observed high levels of MHC (functional) diversity in Mediterranean brown trout populations. Furthermore, under host–parasite coevolution, RAA is expected to cause cyclical fluctuations of MHC variants, hence contributing to generate the differences in MHC allele/supertype frequencies observed among populations (Spurgin & Richardson, 2010). Remarkably, the adopted modeling approach allowed disentangling the relative contribution of RAA and HA, which is notoriously challenging because sufficiently rare variants favored under RAA mostly occur in heterozygotes (Radwan et al., 2020; Spurgin & Richardson, 2010).

In brown trout, the analysis of the relationship between specific MHC functional variants and body condition revealed that individuals carrying the supertype‐7 were generally in better body condition, suggesting that possessing this functional variant may confer a fitness advantage, possibly an improved resistance against pathogens. The opposite could be hypothesized for individuals carrying the supertype‐5 or supertype‐11, although the latter likely reflects local rather than general effects as the vast majority of individuals carrying supertype‐11 occurred in a single population (MEL). Positive and/or negative correlations between fitness‐related traits and certain MHC alleles (reviewed in Sommer, 2005), as well as supertypes (Schwensow et al., 2007; Sepil et al., 2012, 2013), are usually believed evidence of RAA, reflecting a snapshot of the MHC–pathogen cyclical interactions within a population (Spurgin & Richardson, 2010). This could be the case of supertype‐11 in our study. On the other hand, the occurrence of associations in multiple unrelated populations simultaneously (i.e., supertype‐5 and supertype‐7) is not entirely consistent with RAA, since MHC–pathogen dynamics under RAA should be spatially unsynchronized. However, they may be consistent even with other mechanisms of pathogen‐mediated selection, such as fluctuating selection (Radwan et al., 2020; Spurgin & Richardson, 2010). In addition, RAA does not necessarily exclude interactions between specific MHC molecules and pathogens (Trachtenberg et al., 2003). Thus, RAA, possibly along with other mechanisms, could explain the relationship between body condition and the local frequency of MHC variants, as well as its spatially consistent association with certain MHC supertypes. Anyway, further studies accounting for accurate information on parasites or infection status of individuals are needed to clarify the relationship between brown trout MHC supertypes and parasite load.

Our findings overall are mostly in line with RAA expectations, although partially inconsistent with results from previous studies targeting salmonids. For instance, Langefors et al. (2001) identified specific Atlantic salmon MHC alleles associated with resistance/susceptibility to furunculosis caused by *Aeromonas salmonicida* in an experimental infection study, interpreting these outcomes as frequency‐dependent selection (RAA). An analogous conclusion was reached in a study assessing offspring survivorship in the Chinook salmon (Pitcher & Neff, 2006). In both studies, no signal for HA was found: heterozygous individuals were not more resistant than homozygous in the Atlantic salmon, while underdominance was revealed for the Chinook salmon. Evans and Neff (2009) still detected a few MHC alleles associated with susceptibility to bacterial parasites in the Chinook salmon, but MHC class II heterozygous individuals were less infected than homozygous, implying HA. Conversely, *Anisakis* load in wild and artificially crossed Atlantic salmon did not differ between homozygotes and heterozygotes at MHC, with no correlations between specific MHC alleles and resistance to infection, possibly excluding HA and RAA (Consuegra & Garcia De Leaniz, 2008). In contrast to RAA, DAA, and HA expectations, a study orientated toward sexual‐selection revealed that, in *in‐vitro* crosses of brown trout, parents showing low MHC dissimilarity and males with rare MHC genotypes sired embryos with reduced survival, while certain MHC homozygous genotypes produced offspring with higher survival compared to heterozygous ones (Jacob et al., 2010). To our knowledge, DAA has not been explicitly addressed on salmonids to date. Nevertheless, MHC amino acid dissimilarity was more than expected by chance within individuals in a wild population of Atlantic salmon (Consuegra & Garcia De Leaniz, 2008), conversely to a few populations of introduced brown trout and rainbow trout (Monzón‐Argüello et al., 2014): in both cases, authors interpreted the results in the light of disassortative/assortative mating, but it could also indicate occurrence/lack of DAA in our opinion.

### 4.4 Effects of human‐mediated introgression on MHC diversity and conservation remarks

Introgression from domestic lineages was detected in all examined populations, as indicated by the various frequencies of hybrids at the LDH gene. Noticeably, MHC‐DAB richness (as well as *STR richness*) increased with increasing LDH‐based admixture, thus supporting introgression as a non‐negligible source of adaptive diversity in wild populations (Hedrick, 2013). Because adaptive genes undergoing balancing selection (RAA) are predicted to permeate into the gene pool of sink populations more readily than neutral ones (Schierup et al., 2000), introgression, even if limited, should be more pronounced for MHC than neutral markers (Dudek et al., 2019). Such a marker‐dependent pattern qualitatively emerged from the breakdown of AMOVAs (Table 3): the difference between groups of relatively pure and massively stocked populations was substantial although marginally significant for STR (9%, *p* = .063), while reduced for MHC‐DAB (2%, *p* = .069) and negligible for MHC‐ST (< 1%, *p* = .332). Therefore, we may speculate that the relatively recent hybridization together with RAA could have promoted the rapid introgression and the maintenance of hatchery‐introduced, initially rare MHC (functional) variants into native populations, including those only marginally affected by stocking (i.e., SCR and CAR). This may have eventually resulted in an apparently reduced differentiation among roughly pure native and massively stocked populations for MHC, but not for STR. Such hypothesis, however, deserves further investigations.

In a conservation perspective, genetic introgression from hatchery‐reared, non‐autochthonous fish may entail a decreased viability of wild native populations (Bert et al., 2007; Pinter et al., 2019). In the case of adaptive genes, such as MHC, variation may have undergone intentional or unintentional domestication selection during the propagation in captivity, possibly implying nonadaptive evolution due to relaxed pathogen‐mediated selection or, conversely, adaptation induced by a different parasite pressure. According to the latter hypothesis, stronger signals of selection on MHC were observed in hatchery versus wild populations of brown trout in Austria (Schenekar & Weiss, 2017). On the other hand, the maintenance of a broader MHC repertoire within populations may contribute to coping more effectively with novel pathogens, possibly including those introduced by released domestic trout—anyway, if experimentally supported, this would require a careful cost–benefit evaluation. Whatever the actual consequences of anthropogenic‐introduced MHC diversity on brown trout population viability, our study emphasizes the general value of MHC for conservation genetics of wild‐ranging animals. MHC variation, particularly that expressed by functional supertypes, can be highly informative to assess the evolutionary potential and viability of populations because of its relationship with fitness‐related traits (Sommer, 2005; Ujvari & Belov, 2011). Also, MHC may provide a valuable supportive tool for defining conservation and management units (e.g., Zhu et al., 2013). Yet, an integrated conservation approach should take into account both neutral (classical markers) and adaptive (MHC) variation (Sommer, 2005; Ujvari & Belov, 2011), but this is still rarely adopted, unfortunately.

## 5 CONCLUSION

Our study contributes to understanding the evolution of MHC genes in wild populations: we provide evidence for the interplay between neutral (genetic drift, demography) and selective (balancing selection) processes to shape the pattern of adaptive (MHC) diversity in brown trout, disentangling between balancing selection mechanisms actually involved in maintaining MHC (functional) variation. We also demonstrated how human‐mediated introgression with domestic trout coupled with balancing selection may affect patterns of adaptive diversity. Finally, our findings support the power and the biological relevance of the supertyping approach to investigate selection and accurately analyze the functional variation of highly polymorphic genes of MHC (Naugler & Liwski, 2008; Schwensow et al., 2007; Sepil et al., 2012, 2013; Sette & Sidney, 1998; Trachtenberg et al., 2003).

## CONFLICT OF INTEREST

None declared.

## AUTHOR CONTRIBUTIONS

**Lorenzo Talarico:** Conceptualization (lead); formal analysis (equal); investigation (lead); methodology (equal); visualization (equal); writing–original draft (lead); writing–review and editing (lead). **Silvio Marta:** Conceptualization (supporting); formal analysis (equal); methodology (equal); visualization (equal); writing–review and editing (supporting). **Anna Rita Rossi:** Conceptualization (supporting); funding acquisition (supporting); resources (equal); writing–review and editing (supporting). **Simone Crescenzo:** Investigation (equal). **Gerardo Petrosino:** Investigation (equal). **Marco Martinoli:** Investigation (supporting). **Lorenzo Tancioni:** Conceptualization (lead); funding acquisition (lead); investigation (equal); resources (equal); writing–review and editing (supporting).

## Supporting information

Appendix S1‐S10

## Data Availability

The following data have been deposited in Dryad (https://doi.org/10.5061/dryad.xsj3tx9fg): paired‐end Fastq files and individual tag for demultiplexing; individual genetic and phenotypic data; the R script for supertype randomizations; and supplementary information. The novel 41 MHC‐DAB alleles of *Salmo trutta* complex were deposited in GenBank (accessions: MW679462‐MW679502).
