# Supplementary material for: Balancing selection, genetic drift, and human‐mediated introgression interplay to shape MHC (functional) diversity in Mediterranean brown trout
Source: Ecol Evol. 2021 Jul 15;11(15):10026–41. doi: 10.1002/ece3.7760 (PMC8328470; doi:10.1002/ece3.7760)
Supplement: Supplementary file 1 — Appendix S1‐S10 [file ECE3-11-10026-s001.docx]

# Appendices for

**“Balancing selection, genetic drift, and human mediated-introgression interplay to shape MHC (functional) diversity in Mediterranean brown trout”**

*L. Talarico^1^, S. Marta^2^, A. R. Rossi^3^, S. Crescenzo^3^, G. Petrosino^3^, M. Martinoli^1,4^, L. Tancioni^1^*

^1^ Laboratory of experimental Ecology and Aquaculture, Department of Biology, University of Rome “Tor Vergata”, Rome (Italy).

^2^ Department of Environmental Science and Policy, University of Milan, Milan (Italy).

^3^ Department of Biology and Biotechnology C. Darwin, University of Rome “La Sapienza”, Rome (Italy).

^4^ Consiglio per la Ricerca in Agricoltura e l'Analisi dell'Economia Agraria (CREA), Centro di Zootecnia e Acquacoltura, Monterotondo (Italy).

**Appendix S1. Amplification, next-generation amplicon sequencing and genotyping of the MHC-DAB locus.**

A 254-257 bp fragment of exon 2 of the MHC class II DAB gene was amplified using the primer pair CL007 (5’-GATCTGTATTATGTTTTCCTTCCAG-3’) and AL100 (5’-CACCTGTCTTGTCCAGTATG-3’) (Olsén et al., 1998). Primers included also partial Illumina Nextera XT adaptors to allow library generation, and a four-bp tag (five forward and five reverse tagged primers were combined to obtain 25 unique barcode combinations). Each PCR reaction (total volume = 20 μl) contained 20-100 ng DNA, 0.375 μM each primer, 1X Phusion Buffer HF, 200 μM each dNTP, and 0.4 U of Phusion Hot Start II High-Fidelity DNA polymerase (Thermo Scientific – Waltham, MA, USA). Amplifications were carried out in a 2720 Thermal cycler (Applied Biosystems – Foster City, CA, USA) as following: initial denaturation at 98 °C for 2’, followed by 30 cycles at 98 °C for 15”, 52 °C annealing for 20”, 72 °C for 20”, and a final elongation step at 72 °C for 7’. We assembled multiple batches of 6-19 barcoded amplicons each, by pooling amplicons roughly equimolarly, as assessed by visual examination of band intensities on 1% agarose gel – but note that batches included also MHC-DAB amplicons of *Salmo spp*. from another experiment. A commercial company (Bio-Fab Research, Rome, Italy) carried out the preparation of libraries (i.e. adding the remaining part of Illumina adapters and indexes in a second PCR step) and performed a 300 bp paired-end sequencing on Illumina MiSeq (Illumina – San Diego, CA, USA).

AmpliSAT online tools (<http://evobiolab.biol.amu.edu.pl/amplisat/index.php>) were used for reads pre-processing: merging of paired-end reads (default parameters of the FLASH algorithm; Magoč & Salzberg 2011); discarding of reads with a low base call accuracy (i.e. average quality < 25 Phred) and a length out of the range 290-320 bp; removing of primer sequences; de-multiplexing; counting of variants for each amplicon. The overall genotyping procedure consisted of three steps: (1) visual inspection of results with AmpliCHECK to identify artefacts and optimal parameters for the following clustering step; (2) AmpliSAS clustering, to automatically remove a large part of artefacts and contextually increase the depth of putative true variants; (3) filtering of residual artefacts and actual genotyping according to the DOC (Degree Of Change) method. In the first step, we sorted all variants by decreasing maximum per-amplicon frequency (PAF) and observed that variants showing a maximum PAF < 5.3% were mostly identified as chimaeras or low-frequency sequencing/PCR errors (i.e. variants showing 1-2 base mismatches from true parental variants with higher PAF within the same amplicon). Secondly, in the sequence-similarity based clustering performed by AmpliSAS (Sebastian et al., 2016), the above-mentioned detected artefacts (i.e. variants with < 5.3% PAF) were removed and their depth added to similar high-frequency variants within the same amplicon (i.e. parental variants from which artefacts presumably originated). Thirdly, to remove residual artefacts that originated from contaminations and/or tag-jumping, we applied the DOC procedure of Lighten et al. (2014), implemented as a filtering step in AmpliSAS: under the assumption that true variants occur in higher PAF than artefacts, the break point separating true variants from artefacts is set within each amplicon separately by ordering variants by their PAF and finding the highest drop in the cumulative frequency of consecutive variants (i.e. the maximum DOC). Note that the clustering step is performed to improve the accuracy of the DOC genotyping.

## Appendix S2. Microsatellite amplification and genotyping.

A panel of 11 polymorphic microsatellite loci (STR) was chosen from those available from literature (see table below). PCR reactions were conducted in a total volume of 10 µL containing 1 µL of 10X buffer, 0.3 µL of MgCl2 (50 mM), 0.2 µL of dNTP (200 µM), 0.1 µL of each primer (100 µM), 0.07 µL of BIOTAQ^TM^ DNA polymerase (Bioline) and 10–100 ng of DNA template. Forward primers for each locus were labelled with 5′-fluorescent dye (6-FAM, HEX or ATTO550). Amplifications were carried out in a Multigene OptiMax Thermalcycler with an initial denaturation of 2’ at 95°C followed by 30 cycles of 94°C for 30”, a locus-specific annealing temperature (see table below) for 30” and 72°C for 1’ and a final extension at 72°C for 10’. Amplicons were electrophoresed on a 1% agarose gel with ethidium bromide and visualized under UV light to assess successful reactions.

Amplified PCR fragments were then diluted and separated on an ABI3730XL sequencer (Macrogen Inc.) with GeneScan 500 LIZ dye size standards. Allele sizes were determined using the Peak Scanner Software v2.0 (Applied Biosystems).

| **Locus** | **Primer** | **Primer sequence (5' --> 3')** | **Annealing**  **Temp. (°C)** |  | **Reference** |
| --- | --- | --- | --- | --- | --- |
| **STR60** | Forward | CGGTGTGCTTGTCAGGTTTC | 60 |  | Estoup et al., 1993 |
|  | Reverse | GTCAAGTCAGCAAGCCTCAC |  |  |  |
| **STR73** | Forward | CCTGGAGATCCTCCAGCAGGA | 60 |  | Estoup et al., 1993 |
|  | Reverse | CTATTCTGCTTGTAACTGACCTA |  |  |  |
| **SSA85** | Forward | ACCCGCTCCTCACTTAATC | 63 |  | O’Reilly et al., 1996 |
|  | Reverse | AGG TGG GTC CTC CAA GCT AC |  |  |  |
| **SSOSL417** | Forward | TTGTTCAGTGTATATGTGTCCCAT | 53 |  | Slettan et al., 1995 |
|  | Reverse | GATCTTCACTGCCACCTTATGACC |  |  |  |
| **SSA410UOS** | Forward | GGAAAATAATCAATGCTGCTGGTT | 60 |  | Cairney et al., 2000 |
|  | Reverse | CTACAATCTGGACTATCTTCTTCA |  |  |  |
| **SSA408UOS** | Forward | AATGGATTACGGGTA CGT TAGACA | 62 |  | Cairney et al., 2000 |
|  | Reverse | CTCTTGTGCAGGTTCTTC ATCTGT |  |  |  |
| **SSSP2213** | Forward | ATGTGGAGGTCAACTAACCAGCGTG | 58 |  | Paterson et al., 2004 |
|  | Reverse | CATCAATCACAGAGTGAGGCACTCG |  |  |  |
| **OMM1064** | Forward | AGAATGCTACTGGTGGCTGTATTGTGA | 60 |  | Rexroad et al., 2002 |
|  | Reverse | TCTGAAAGCAGGTGGATGGTTCC |  |  |  |
| **SSAD190** | Forward | GGCATTGGAGGTAAGGACAC | 60 |  | King et al., 2005 |
|  | Reverse | CCAGACCACTGAACTTCTCATC |  |  |  |
| **ONEPHI2** | Forward | GGTGCCAAGGTTCAGTTTATGTT | 60 |  | Scribner et al., 1996 |
|  | Reverse | CAGGAATTTACAGGACCCAGGTT |  |  |  |
| **SSA103NVH** | Forward | GCTGTGATTTCTCTCTGC | 58 |  | Thorsen et al., 2005 |
|  | Reverse | AAAGGTGGGTCCAAGGAC |  |  |  |

## Appendix S3. Bayesian analysis of population structure.

***Methods***

The Bayesian clustering implemented in STRUCTURE v.2.3.4 (Pritchard et al., 2000) was used to identify genetic groups based on 11 microsatellites loci. Analysis settings were as follow: admixture model with correlated allele frequencies; five repeated runs for each K value in the range 1-8; 400,000 iterations after a burn-in period of 200,000. To define the optimal number of K we choose the highest ΔK value (Evanno et al., 2005). Finally, we combined the results of multiple runs for the chosen optimal K in CLUMPAK (Kopelman et al., 2015).

***Results***

Six genetic clusters were clearly supported according to the Evanno’s method (Fig. A), each corresponding to a unique population. Populations showed low admixture degree (range: 2% - 18%), hence indicating that they were roughly genetically isolated from each others.


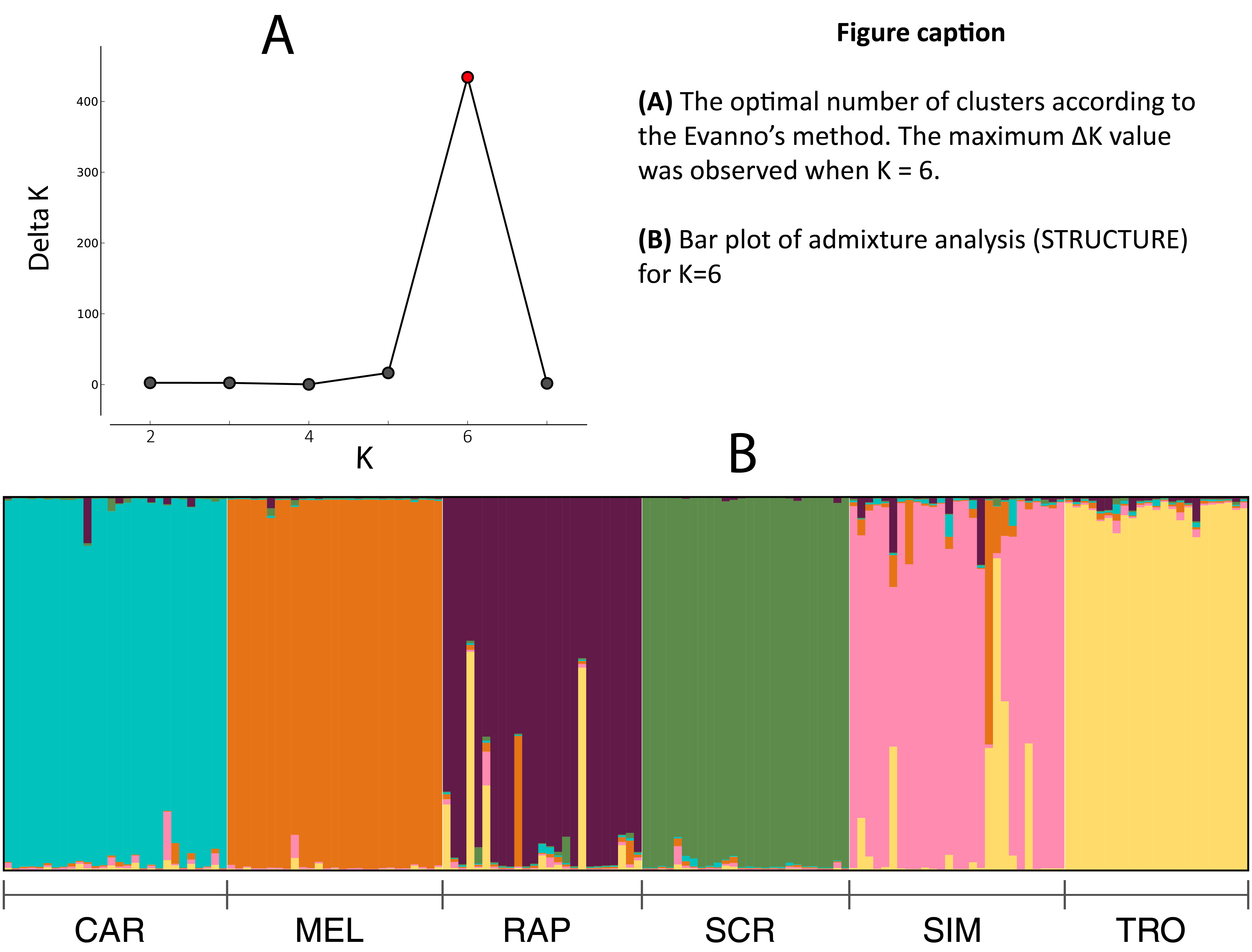


## Appendix S4. Amino acid alignment and PSS in 58 brown trout MHC-DAB alleles.

Amino acid alignment of 58 MHC-DAB alleles of *S. trutta* found in overall 156 brown trout individuals*.* Plus signs (+) mark sites under positive selection (PSSs) detected by a particular test, i.e. SLAC, FEL, REL, MEME, FUBAR, BEB (p = p-value; PP = posterior probability; BF = Bayes factor) and corresponding antigen-binding sites (ABS) in human MHC (Brown et al., 1993). Salmon-shaded sites highlight PSSs assumed in this study, i.e. those detected by at least two tests. The grey-shaded site denotes the recombination breakpoint detected by the SBP analysis. Note that alleles MHC-DAB*078 - *118 have been detected for the first time in this study.


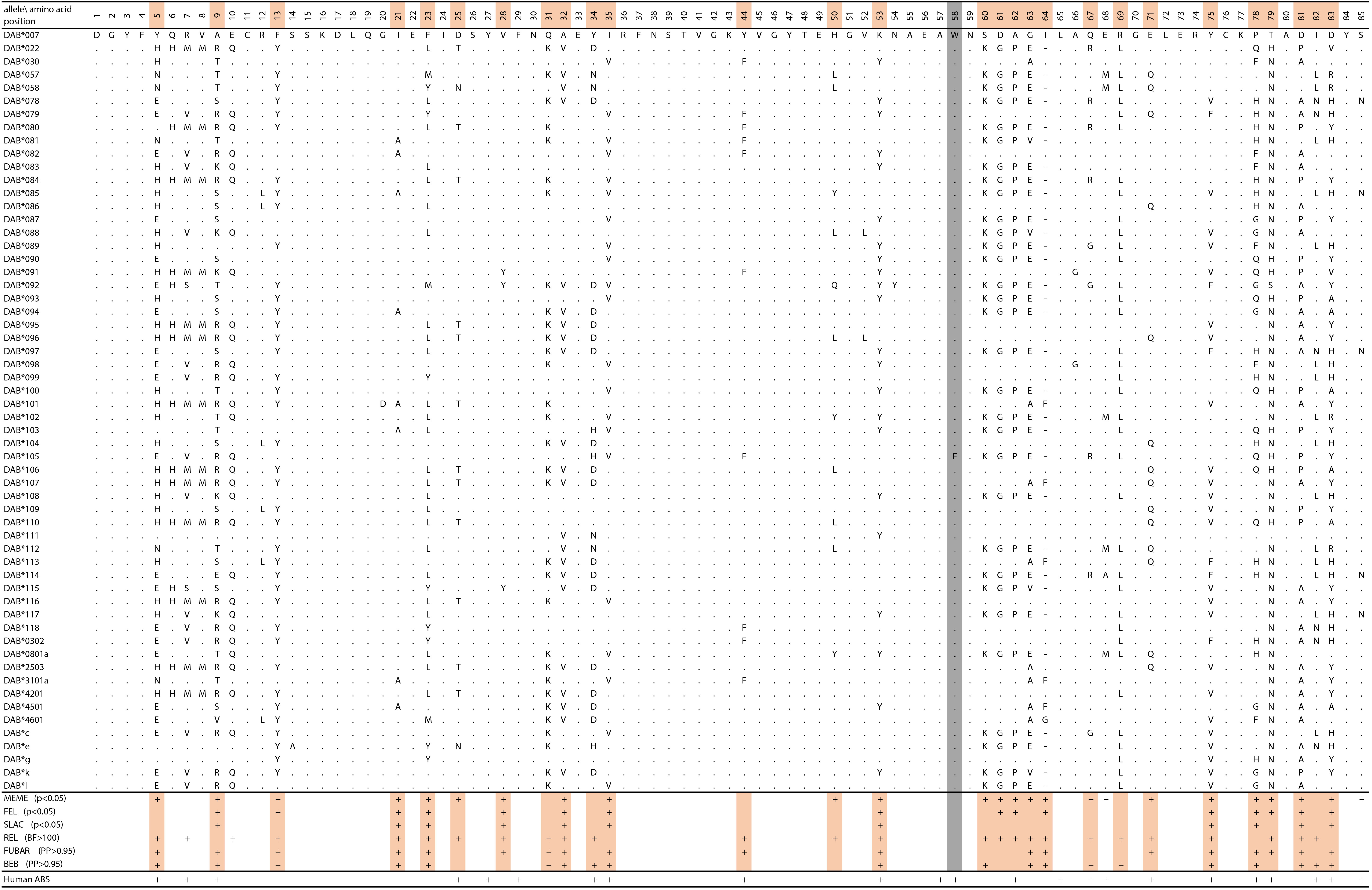


## **Appendix S5. Frequency MHC alleles and supertypes across populations**.

Frequencies of 58 MHC-DAB alleles and 12 supertypes in six Mediterranean brown trout populations. Alleles are listed according to supertype grouping; GenBank accessions are given in parentheses for alleles already detected in previous studies; supertype frequencies are shown in bold-italic.

| **MHC-DAB allele** | **MHC supertype** | **CAR** | **MEL** | **RAP** | **SCR** | **SIM** | **TRO** |
| --- | --- | --- | --- | --- | --- | --- | --- |
| *DAB*007 (DQ257393)* | 1 | 0.036 | - | - | 0.019 | 0.074 | - |
| *DAB*111* | 1 | - | - | - | - | - | 0.022 |
|  |  | ***0.036*** | ***-*** | ***-*** | ***0.019*** | ***0.074*** | ***0.022*** |
| *DAB*081* | 2 | - | - | 0.260 | - | 0.019 | - |
| *DAB*085* | 2 | - | - | - | 0.231 | - | - |
| *DAB*089* | 2 | - | - | 0.080 | - | - | - |
| *DAB*102* | 2 | - | 0.019 | - | - | - | 0.022 |
| *DAB*c (HM596879)* | 2 | 0.375 | - | - | - | 0.037 | - |
| *DAB*e (HM596880)* | 2 | 0.036 | 0.019 | 0.060 | - | 0.037 | - |
| *DAB*0801a (KT003665)* | 2 | 0.018 | 0.074 | - | - | 0.019 | 0.043 |
|  |  | ***0.429*** | ***0.111*** | ***0.400*** | ***0.231*** | ***0.111*** | ***0.065*** |
| *DAB*104* | 3 | - | - | - | - | 0.019 | 0.043 |
| *DAB*113* | 3 | - | - | 0.020 | - | - | - |
| *DAB*4501 (KT003653)* | 3 | - | 0.037 | - | - | 0.037 | - |
| *DAB*4601 (KT003655)* | 3 | - | - | - | - | 0.019 | - |
|  |  | ***-*** | ***0.037*** | ***0.020*** | ***-*** | ***0.074*** | ***0.043*** |
| *DAB*087* | 4 | - | - | 0.080 | - | - | 0.043 |
| *DAB*090* | 4 | - | - | - | - | - | 0.087 |
| *DAB*093* | 4 | - | - | - | - | 0.056 | - |
| *DAB*100* | 4 | - | - | - | - | - | 0.065 |
| *DAB*103* | 4 | - | - | 0.020 | - | 0.019 | 0.022 |
| *DAB*105* | 4 | - | - | - | - | - | 0.043 |
|  |  | ***-*** | ***-*** | ***0.100*** | ***-*** | ***0.074*** | ***0.261*** |
| *DAB*022 (DQ257408)* | 5 | - | 0.130 | - | - | 0.019 | - |
| *DAB*080* | 5 | 0.054 | - | - | - | 0.315 | - |
| *DAB*084* | 5 | 0.214 | - | - | - | - | - |
|  |  | ***0.268*** | ***0.130*** | ***-*** | ***-*** | ***0.333*** | ***-*** |
| *DAB*078* | 6 | - | - | 0.300 | - | 0.019 | 0.239 |
| *DAB*094* | 6 | - | - | - | - | 0.074 | - |
| *DAB*097* | 6 | - | - | - | - | - | 0.043 |
| *DAB*114* | 6 | 0.018 | - | - | - | - | - |
| *DAB*115* | 6 | 0.018 | - | - | - | - | - |
| *DAB*k (HM596885)* | 6 | - | - | 0.040 | 0.019 | - | 0.022 |
|  |  | ***0.036*** | ***-*** | ***0.340*** | ***0.019*** | ***0.093*** | ***0.304*** |
| *DAB*030 (DQ491032)* | 7 | - | - | - | - | 0.056 | - |
| *DAB*082* | 7 | 0.018 | - | - | 0.308 | - | - |
| *DAB*086* | 7 | - | 0.111 | - | - | - | - |
| *DAB*091* | 7 | 0.071 | - | - | - | - | - |
| *DAB*109* | 7 | - | - | - | - | - | 0.022 |
| *DAB*3101a (KT003662)* | 7 | - | - | - | - | 0.019 | - |
|  |  | ***0.089*** | ***0.111*** | ***-*** | ***0.308*** | ***0.074*** | ***0.022*** |
| *DAB*095* | 8 | - | 0.019 | - | - | 0.019 | - |
| *DAB*096* | 8 | - | - | - | - | - | 0.043 |
| *DAB*101* | 8 | - | - | - | - | - | 0.065 |
| *DAB*106* | 8 | - | - | 0.040 | - | - | - |
| *DAB*107* | 8 | - | - | - | - | 0.019 | - |
| *DAB*110* | 8 | - | - | - | - | 0.019 | - |
| *DAB*116* | 8 | 0.018 | - | - | - | - | - |
| *DAB*2503 (KT003645)* | 8 | - | - | - | - | 0.019 | - |
| *DAB*4201 (KT003648)* | 8 | - | - | - | - | 0.019 | - |
|  |  | ***0.018*** | ***0.019*** | ***0.040*** | ***-*** | ***0.093*** | ***0.109*** |
| *DAB*083* | 9 | - | - | - | 0.327 | - | - |
| *DAB*088* | 9 | 0.036 | - | - | 0.038 | - | - |
| *DAB*108* | 9 | - | - | 0.020 | - | - | - |
| *DAB*117* | 9 | - | - | - | - | - | 0.022 |
| *DAB*l (HM596886)* | 9 | - | - | - | - | - | 0.087 |
|  |  | ***0.036*** | ***-*** | ***0.020*** | ***0.365*** | ***-*** | ***0.109*** |
| *DAB*057 (DQ491059)* | 10 | - | 0.037 | - | - | - | 0.022 |
| *DAB*058 (DQ491060)* | 10 | - | 0.167 | - | - | 0.019 | 0.022 |
| *DAB*112* | 10 | - | - | - | - | - | 0.022 |
|  |  | ***-*** | ***0.204*** | ***-*** | ***-*** | ***0.019*** | ***0.065*** |
| *DAB*079* | 11 | - | 0.333 | 0.020 | - | 0.019 | - |
| *DAB*098* | 11 | - | - | - | 0.058 | - | - |
| *DAB*099* | 11 | - | 0.019 | - | - | 0.019 | - |
| *DAB*118* | 11 | - | - | - | - | 0.019 | - |
| *DAB*0302 (KT003651)* | 11 | - | 0.037 | - | - | - | - |
| *DAB*g (HM596882)* | 11 | 0.089 | - | - | - | - | - |
|  |  | ***0.089*** | ***0.389*** | ***0.020*** | ***0.058*** | ***0.056*** | ***-*** |
| *DAB*092* | 12 | - | - | 0.060 | - | - | - |
|  |  | ***-*** | ***-*** | ***0.060*** | ***-*** | ***-*** | ***-*** |

## Appendix S6. Results of PAML analyses.

Evaluation of the goodness of fit for three models of codon evolution and estimated parameter values as performed in PAML. ω, the dN/dS ratio; p_1_, the proportion of positively selected sites (ω> 1); ω_1_, the estimated value of ω for sites under positive selection; ΔAIC, the difference between the value of the Akaike information criterion (AIC) of a given model and the best model.

| *Model* | *ΔAIC* | *Parameters* |
| --- | --- | --- |
| M0 — one ω | 641.7 | ω = 3.613 |
| M7 — nearly neutral with beta | 277.5 |  |
| M8 — positive selection with beta (ω_0_ ≤ 1. ω_1_ > 1) | best | p_1_ = 0.257; ω_1_ = 9.213 |

## Appendix S7. Estimates of null alleles.

Estimated frequencies of null alleles for each marker (11 microsatellite loci and the MHC-DAB locus) and population according to the Dempster’s EM algorithm.

| ***Pop\Locus*** | ***STR60*** | ***STR73*** | ***SSOSL417*** | ***SSA408UOS*** | ***OMM_1064*** | ***SSAD190*** | ***SSSP2213*** | ***SSA85*** | ***ONEPHI2*** | ***SSA410UOS*** | ***SSA413NVH*** | ***MHC-DAB*** |
| --- | --- | --- | --- | --- | --- | --- | --- | --- | --- | --- | --- | --- |
| ***CAR*** | 0.00 | 0.00 | 0.00 | 0.04 | 0.00 | 0.00 | 0.03 | 0.00 | 0.09 | 0.02 | 0.00 | 0.00 |
| ***MEL*** | 0.00 | 0.00 | 0.00 | 0.01 | 0.00 | 0.00 | 0.04 | 0.01 | 0.14 | 0.01 | 0.00 | 0.00 |
| ***RAP*** | 0.00 | 0.00 | 0.00 | 0.00 | 0.00 | 0.00 | 0.00 | 0.00 | 0.04 | 0.00 | 0.00 | 0.00 |
| ***SCR*** | 0.00 | 0.00 | 0.00 | 0.00 | 0.04 | 0.00 | 0.00 | 0.00 | 0.11 | 0.00 | 0.00 | 0.03 |
| ***SIM*** | 0.00 | 0.00 | 0.04 | 0.00 | 0.00 | 0.04 | 0.00 | 0.00 | 0.03 | 0.00 | 0.00 | 0.10 |
| ***TRO*** | 0.00 | 0.03 | 0.00 | 0.00 | 0.01 | 0.00 | 0.00 | 0.02 | 0.03 | 0.01 | 0.01 | 0.00 |

## Appendix S8. Hardy-Weinberg equilibrium tests.

Results of exact tests for Hardy-Weinberg equilibrium in six *Salmo trutta* populations. Holm-Bonferroni adjusted p-values are provided for 11 microsatellites loci and the MHC. Statistically significant values (p < 0.05) are in bold.

| **Pop\Locus** | **STR60** | **STR73** | **SSOSL417** | **SSA408UOS** | **OMM_1064** | **SSAD190** | **SSSP2213** | **SSA85** | **ONEPHI2** | **SSA410UOS** | **SSA413NVH** | **MHC-DAB** |
| --- | --- | --- | --- | --- | --- | --- | --- | --- | --- | --- | --- | --- |
| **CAR** | 1 | 1 | 0.77 | 0.86 | 1 | 1 | 1 | 1 | 0.63 | 1 | - | 0.64 |
| **MEL** | 1 | 1 | 0.68 | 1 | 1 | 1 | 1 | 0.69 | 0.12 | 1 | 1 | 0.64 |
| **RAP** | - | 0.48 | **0.01** | 0.70 | 0.60 | 0.92 | 1 | 0.07 | 0.12 | 1 | 1 | 0.25 |
| **SCR** | 1 | 1 | 0.77 | 1 | 1 | 1 | 1 | 0.69 | 0.63 | 1 | 1 | 0.64 |
| **SIM** | 0.22 | 1 | 0.77 | 1 | 1 | 1 | 1 | 0.69 | 0.85 | 0.09 | 1 | **0.01** |
| **TRO** | 1 | 1 | 0.77 | 1 | 0.99 | 1 | 1 | 1 | 0.85 | 1 | 0.59 | 0.64 |

## Appendix S9. Population pairwise genetic distances

Matrices of genetic distance between six Mediterranean brown trout populations based on 11 microsatellite loci (STR), the MHC-DAB locus and MHC supertypes (MHC-ST). Pairwise G_ST_ values and their Bonferroni-corrected significance (obtained with 9999 permutations) are given below and above the diagonal, respectively. ** = p ≤ 0.01.

| ***STR*** | **CAR** | **MEL** | **RAP** | **SCR** | **SIM** | **TRO** |
| --- | --- | --- | --- | --- | --- | --- |
| **CAR** | - | ** | ** | ** | ** | ** |
| **MEL** | 0.292 | - | ** | ** | ** | ** |
| **RAP** | 0.267 | 0.101 | - | ** | ** | ** |
| **SCR** | 0.394 | 0.365 | 0.330 | - | ** | ** |
| **SIM** | 0.201 | 0.128 | 0.114 | 0.309 | - | ** |
| **TRO** | 0.215 | 0.114 | 0.082 | 0.330 | 0.083 | - |

| ***MHC-DAB*** | **CAR** | **MEL** | **RAP** | **SCR** | **SIM** | **TRO** |
| --- | --- | --- | --- | --- | --- | --- |
| **CAR** | - | ** | ** | ** | ** | ** |
| **MEL** | 0.177 | - | ** | ** | ** | ** |
| **RAP** | 0.179 | 0.158 | - | ** | ** | ** |
| **SCR** | 0.213 | 0.203 | 0.205 | - | ** | ** |
| **SIM** | 0.119 | 0.120 | 0.124 | 0.173 | - | ** |
| **TRO** | 0.135 | 0.113 | 0.049 | 0.160 | 0.084 | - |

| ***MHC-ST*** | **CAR** | **MEL** | **RAP** | **SCR** | **SIM** | **TRO** |
| --- | --- | --- | --- | --- | --- | --- |
| **CAR** | - | ** | ** | ** | ** | ** |
| **MEL** | 0.131 | - | ** | ** | ** | ** |
| **RAP** | 0.103 | 0.207 | - | ** | ** | ** |
| **SCR** | 0.140 | 0.178 | 0.186 | - | ** | ** |
| **SIM** | 0.057 | 0.099 | 0.134 | 0.162 | - | ** |
| **TRO** | 0.182 | 0.179 | 0.079 | 0.168 | 0.097 | - |

## Appendix S10. Results of randomizations for pairwise population G_ST_ based on MHC supertypes

The observed (green dots) and simulated (blue dots) genetic differentiation (Nei’s G_ST_) based on MHC supertypes. The average per-population simulated values and their 95% confidence intervals (blue bars) were obtained with 10000 randomizations in which MHC alleles were shuffled into 12 groups of the size of supertype groupings. Observed G_ST_ do not significantly differ from null expectations (p > 0.05). The R script for this analysis is available in Dryad (DOI <https://doi.org/10.5061/dryad.xsj3tx9fg> ).


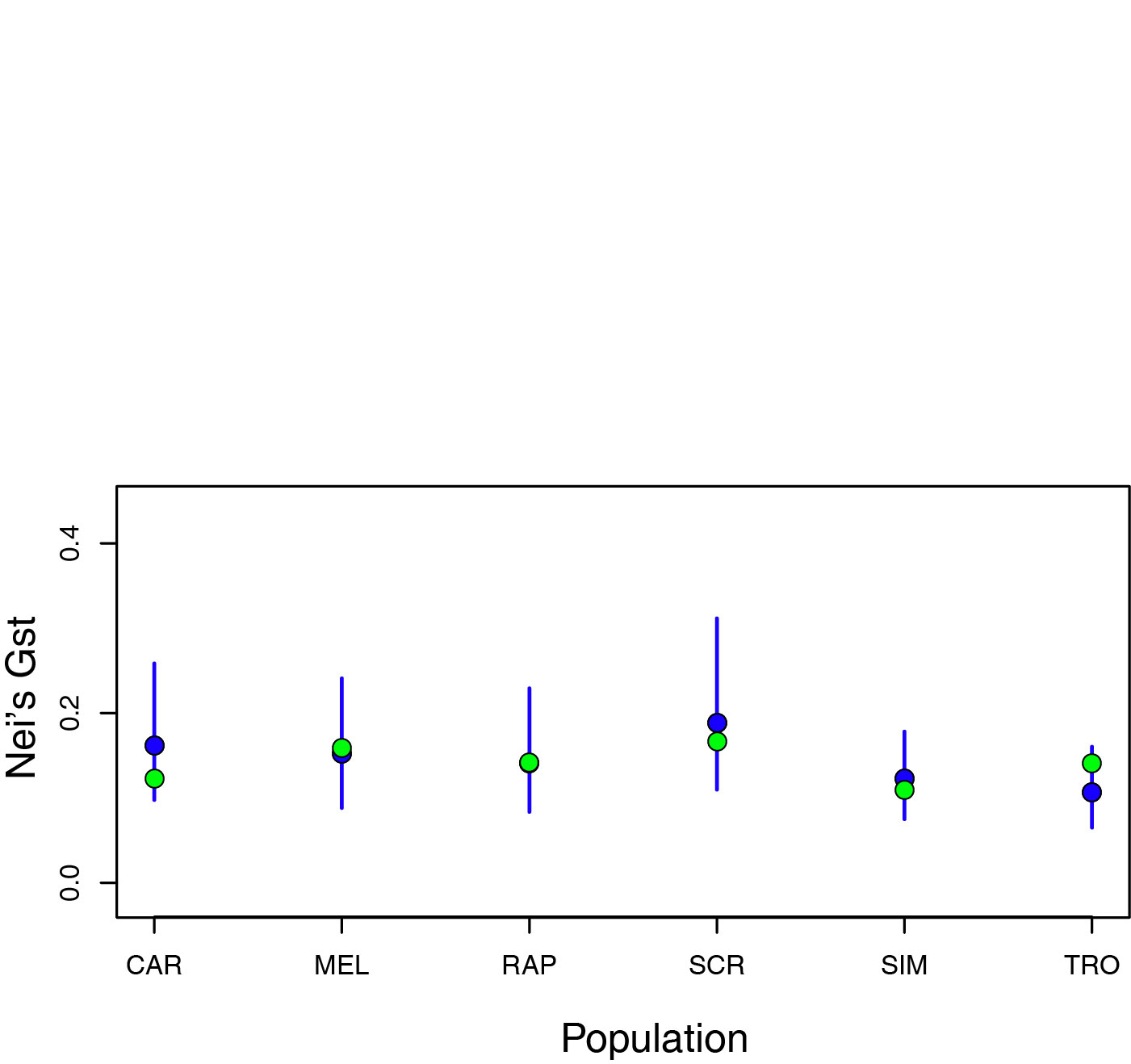


# References of Appendices

Brown, J. H., Jardetzky, T. S., Gorga, J. C., Stern, L. J., Urban, R. G., Strominger, J. L., & Wiley, D. C. (1993). Three-dimensional structure of the human class II histocompatibility antigen HLA-DR1. *Nature* *364*(6432), 33–39.

Cairney, M., Taggart, J. B., & Høyheim, B. (2000). Characterization of microsatellite and minisatellite loci in Atlantic salmon (*Salmo salar* L.) and cross-species amplification in other salmonids. *Molecular Ecology*, *9*(12), 2175–2178.

Estoup, A., Presa, P., Krieg, F., Vaiman, D., & Guyomard, R. (1993). (CT)n and (GT)n microsatellites: A new class of genetic markers for *Salmo trutta* L. (brown trout). *Heredity*, *71*(5), 488–496.

Evanno, G., Regnaut, S., & Goudet, J. (2005). Detecting the number of clusters of individuals using the software STRUCTURE : a simulation study. *Molecular Ecology*, *14*, 2611–2620.

King, T. L., Eackles, M. S., & Letcher, B. H. (2005). Microsatellite DNA markers for the study of Atlantic salmon (*Salmo salar*) kinship, population structure, and mixed-fishery analyses. *Molecular Ecology Notes*, *5*(1), 130–132.

Kopelman, N. M., Mayzel, J., Jakobsson, M., Rosenberg, N. A., & Mayrose, I. (2015). CLUMPAK: A program for identifying clustering modes and packaging population structure inferences across K. *Molecular Ecology Resources*, *15*(5), 1179–1191.

Lighten, J., van Oosterhout, C., Paterson, I. G., Mcmullan, M., & Bentzen, P. (2014). Ultra-deep Illumina sequencing accurately identifies MHC class IIb alleles and provides evidence for copy number variation in the guppy (*Poecilia reticulata*). *Molecular Ecology Resources*, *14*(4), 753–767.

Magoč, T., & Salzberg, S. L. (2011). FLASH: Fast length adjustment of short reads to improve genome assemblies. *Bioinformatics*, *27*(21), 2957–2963.

O’Reilly, P. T., Hamilton, L. C., McConnell, S. K., & Wright, J. M. (1996). Rapid analysis of genetic variation in Atlantic salmon (*Salmo salar*) by PCR multiplexing of dinucleotide and tetranucleotide microsatellites. *Canadian Journal of Fisheries and Aquatic Sciences*, *53*(10), 2292–2298.

Olsén, K., Grahn, M., Lohm, J., & Langefors, Å. (1998). MHC and kin discrimination in juvenile Arctic charr, *Salvelinus alpinus* (L.). *Animal Behaviour*, *56*(2), 319–327.

Paterson, S., Piertney, S. B., Knox, D., Gilbey, J., & Verspoor, E. (2004). Characterization and PCR multiplexing of novel highly variable tetranucleotide Atlantic salmon (*Salmo salar* L.) microsatellites. *Molecular Ecology Notes*, *4*(2), 160–162.

Pritchard, J. K., Stephens, M., & Donnelly, P. (2000). Inference of population structure using multilocus genotype data. *Genetics*, *155*(2), 945–959.

Rexroad, C. E., Coleman, R. L., Hershberger, W. K., & Killefer, J. (2002). Rapid communication: Thirty-eight polymorphic microsatellite markers for mapping in rainbow trout. *Journal of Animal Science*, *80*, 541–542.

Scribner, K. T., Gust, J. R., & Fields, R. L. (1996). Isolation and characterization of novel salmon microsatellite loci: Cross-species amplification and population genetic applications. *Canadian Journal of Fisheries and Aquatic Sciences*, *53*(4), 833–841.

Sebastian, A., Herdegen, M., Migalska, M., & Radwan, J. (2016). AMPLISAS: A web server for multilocus genotyping using next-generation amplicon sequencing data. *Molecular Ecology Resources*, *16*(2), 498–510.

Slettan, A., Olsaker, I., & Lie, Ø. (1995). Atlantic salmon, *Salmo salar*, microsatellites at the SSOSL25, SSOSL85, SSOSL311, SSOSL417 loci. *Animal Genetics*, *26*(4), 277–285.

Thorsen, J., Zhu, B., Frengen, E., Osoegawa, K., de Jong, P. J., Koop, B. F., Davidson, W. S. & Høyheim, B. (2005). A highly redundant BAC library of Atlantic salmon (*Salmo salar*): An important tool for salmon projects. *BMC Genomics*, *6*, 50.
